# Supplementary figures and images for: Reliability and validity of the Japanese version of the Paediatric Pain Profile for children with severe motor and intellectual disabilities
Source: PLoS One. 2020 Dec 22;15(12):e0243566. doi: 10.1371/journal.pone.0243566 (PMC7755203; doi:10.1371/journal.pone.0243566)

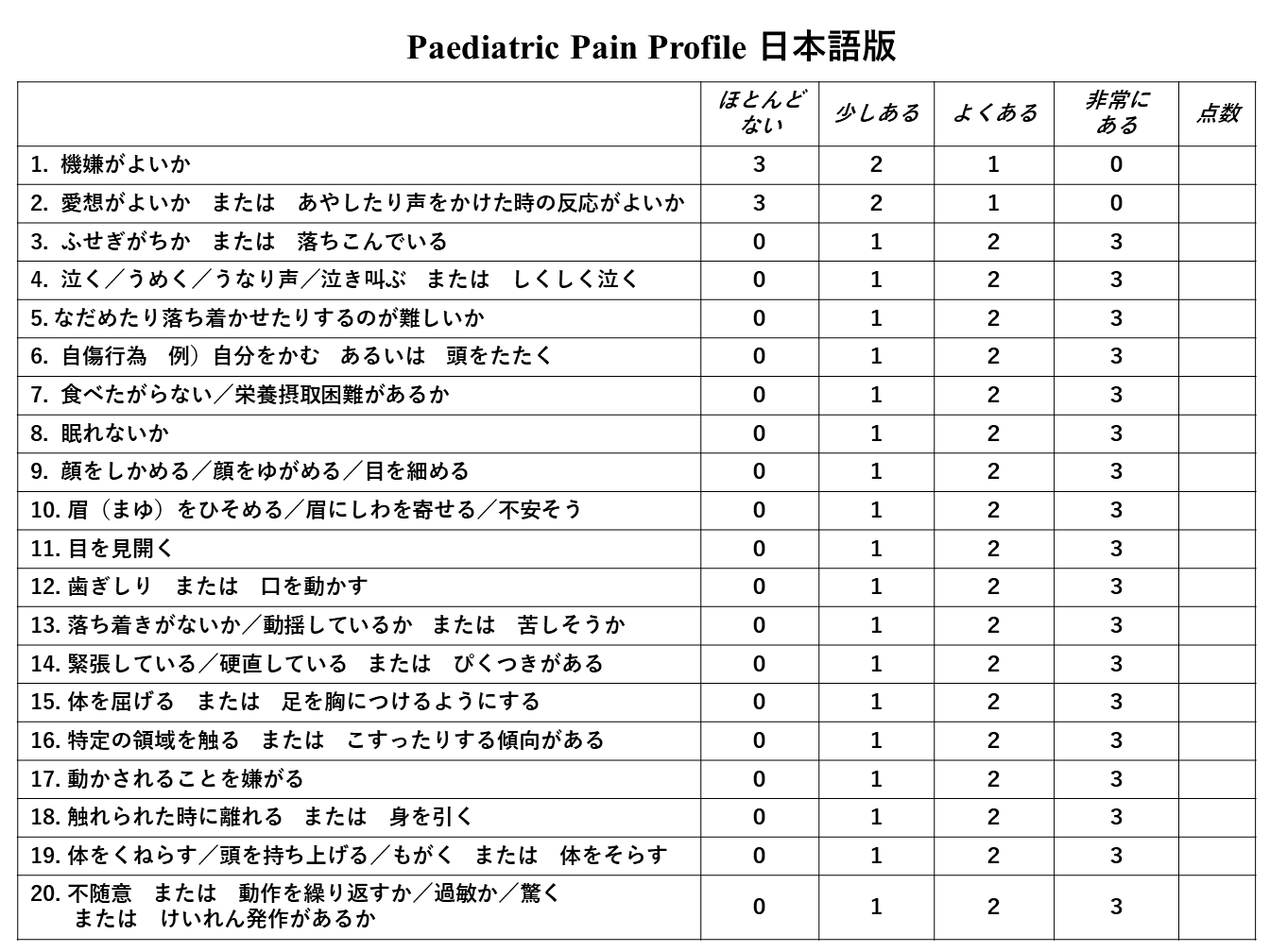

Supplement: S1 Fig — (TIF) [file pone.0243566.s001.tif]

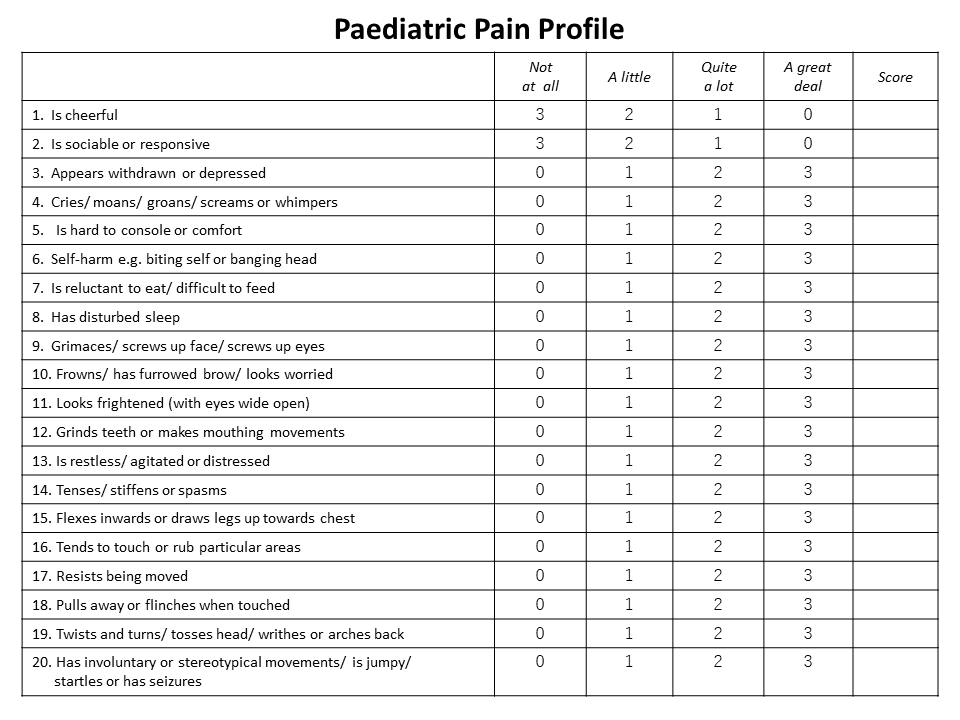

Supplement: S2 Fig — (TIF) [file pone.0243566.s002.tif]
